# Mutagenicity in a Molecule: Identification of Core Structural Features of Mutagenicity Using a Scaffold Analysis

**DOI:** 10.1371/journal.pone.0148900

**Published:** 2016-02-10

**Authors:** Kuo-Hsiang Hsu, Bo-Han Su, Yi-Shu Tu, Olivia A. Lin, Yufeng J. Tseng

**Affiliations:** 1 Graduate Institute of Biomedical Electronics and Bioinformatics, National Taiwan University, Taipei, Taiwan; 2 Department of Computer Science and Information Engineering, National Taiwan University, Taipei, Taiwan; Shantou University Medical College, CHINA

## Abstract

With advances in the development and application of Ames mutagenicity *in silico* prediction tools, the International Conference on Harmonisation (ICH) has amended its M7 guideline to reflect the use of such prediction models for the detection of mutagenic activity in early drug safety evaluation processes. Since current Ames mutagenicity prediction tools only focus on functional group alerts or side chain modifications of an analog series, these tools are unable to identify mutagenicity derived from core structures or specific scaffolds of a compound. In this study, a large collection of 6512 compounds are used to perform scaffold tree analysis. By relating different scaffolds on constructed scaffold trees with Ames mutagenicity, four major and one minor novel mutagenic groups of scaffold are identified. The recognized mutagenic groups of scaffold can serve as a guide for medicinal chemists to prevent the development of potentially mutagenic therapeutic agents in early drug design or development phases, by modifying the core structures of mutagenic compounds to form non-mutagenic compounds. In addition, five series of substructures are provided as recommendations, for direct modification of potentially mutagenic scaffolds to decrease associated mutagenic activities.

## Introduction

In drug discovery, mutagenicity is an issue that needs to be avoided. The detection of mutagenicity at preclinical drug discovery stages could halt the development of potentially harmful drugs and aid in the development of safe therapeutic agents. Mutagenicity is a term used to broadly describe the property of chemical agents or drug substances to induce genetic mutation. It is sometimes used interchangeably with the term genotoxicity, especially concerning the discussion of chemical agents to deleteriously change the genetic material in a cell. However, while all mutagens are genotoxic, not all of the genotoxic substances are mutagenic.[1] To avoid mutagens in the drug candidate screening processes, many efforts have been made in determining mutagenicity of various compounds via *in vitro* approaches, of which the Ames test is the most common.

The Ames test was first introduced in the early 1970’s by Bruce Ames.[2–4] It is a well-established and widely accepted method to assess the mutagenic potential of compounds to cause genetic damage in bacterial cells, for example through frameshift mutation or mutation by base-pair substitution.[2] It is recognized that genetic events are central to the overall development of cancer. Therefore evidence of mutagenic activity may indicate that a chemical substance has the potential to encourage carcinogenic effects. In therapeutic agents, carcinogenicity is strongly correlated with mutagenicity.[5] A positive Ames test would indicate that the chemical is mutagenic and highly likely to be carcinogenic, however false-positive and false-negative test results have been reported as well. Despite that, Ames test is still preferred over the standard *in-vivo* assays, because it provides a quick, convenient, and cost-effective way to estimate the mutagenicity (carcinogenicity) of a compound.

The Ames test has been in use for almost 40 years; the assayed outcome usually correlates with life-time rodent carcinogenicity studies which require 2 years to complete.[6] For the purpose of this study, we mainly focus on the scaffold analysis of DNA reactive (mutagenic) chemical agents in general; therefore the carcinogenic risks associated with these agents will not be discussed. In this study, the word “scaffold” is used primarily to describe the core structure of compounds. In accordance with the International Conference on Harmonisation (ICH) M7 guideline updated in June of 2014, an expert rule-based and statistic-based quantitative structure-activity relationship (QSAR) model can be utilized to estimate the potential mutagenicity of impurities in pharmaceuticals.[7] These models can also be utilized to determine the mutagenicity potential of drugs in safety evaluation. The application of *in silico* models to predict mutagenicity of compounds has been popular in early drug discovery and development processes, sometimes before compounds were synthesized.[8] The time and cost of drug design can be considerably reduced by avoiding to synthesize and analyze compounds with mutagenicity. In recent years, several commercially and publicly available *in silico* tools have been developed to predict the mutagenicity of compounds based on the endpoints of Ames test.

Currently, structural alert-based[9, 10] and QSAR-based[11, 12] models are the two main Ames mutagenicity prediction strategies; users could derive structure-activity relationship and/or mechanistic information from their predictions. Both DEREK for Windows[9] (DfW) and Toxtree[10] are expert prediction systems that utilize structural alerts (SAs) to predict mutagenicity of compounds. The toxicological alerts are derived from literature, academic and industry experts, available experimental data[13–15], and Benigni-Bossa rules.[16] The QSAR-based approaches (e.g., Leadscope Model Applier (LSMA)[11] and MultiCASE (MC4PC)[12]) use regression models to illustrate the relationship between molecular properties (e.g., lipophilicity, polarizability, electron density, and topology) and mutagenicity of compounds being studied.[17] It would be especially useful to be able to correspond the relationship between different core structures of a compound with their associated Ames mutagenicity. However, neither structural alerts nor correlative QSAR-based models can directly indicate whether a scaffold would be more likely to link to mutagenicity.[18] The structural alerts approach only evaluates functional groups and the correlative QSAR-based approach mostly emphasizes on side chain or functional group analysis of an analog series, core structures or scaffolds are not the focus in both approaches. If mutagenicity arises from the scaffold (core structure) itself, these approaches will not be able to flag the scaffold as the major cause of the mutagenic potency. This presents a serious problem because drug compounds are usually constituent from one or several similar core structures with different combinations of side chains. Essentially, all of the drugs from this series might be mutagenic.

In this study, we analyzed the relationship between scaffolds of diverse compounds by correlating the scaffolds and mutagenicity from a dataset of Ames assay for 6,512 compounds collected from literatures.[19] The Scaffold Hunter[20] strategy was adopted to generate hierarchical relationships of scaffolds between these compounds. From analyzing scaffold relationships, we established a list of scaffolds with potential mutagenicity. These scaffolds can be used as a basis for drug design to prevent the development of potentially mutagenic therapeutic agents; they can also be used to suggest non-mutagenic scaffolds to replace mutagenic core structures.

## Materials and Methods

### Benchmark Data Set: Ames Mutagenicity

In recent years, data on Ames mutagenicity have been collected and well organized. The Ames mutagenicity benchmark data set from Hansen[19] includes mutagenicity data collected prior to 2009 and was used in our study. Several recent works[21–23] have also used Hansen’s data set because of its reliability. The Hansen benchmark data set[19] was derived from CCRIS[24], Helma et al.[25], Kazius et al.[26], Feng et al.[27], VITIC[28], and GeneTox[29]. Inorganic molecules and duplicate structures were omitted. Compounds with experimental results that contradicted DEREK or MultiCASE internal data were also removed. Chemical Abstracts Service (CAS) numbers and World Drug Index (WDI) names are provided. The final data set is balanced, containing 3053 mutagens and 3009 non-mutagens (6512 compounds in total). The mean molecular weight is 248 ± 134 (Median MW: 229). An overview is presented in **Table 1**. Due to overlap between different sources, the total amount of relevant data in the individual databases may be higher.

**Table 1:** Overview of the number of compounds in our collected dataset.

	Mutagenic	Non-mutagenic	Total
**CCRIS[24]**	1359	1180	2539
**Kazius et al.[26]**	1375	849	2224
**Helma et al.[25]**	81	57	138
**Feng et al.[27]**	280	111	391
**VITIC[28]**	386	808	1194
**GeneTox[29]**	22	4	26
**Total**	3053	3009	6512

The results were obtained from each source when extending the Ames mutagenicity data set in a stepwise manner.

### Scaffold Hunter

Scaffold Hunter is an interactive tool for intuitive hierarchical structuring, visualization and analysis of complex structure and bioactivity data as well as for the navigation and exploration of chemical space. The program extracts chemically meaningful compound scaffolds (all carbo- and heterocyclic rings, aliphatic linkers and atoms attached via double bonds) from a data set by removing all side chains except exocyclic or linking double bonds. Scaffold Hunter then iteratively removes one ring at a time from larger “parent” scaffolds to yield smaller “child” scaffolds according to the pruning rules [20]. Hierarchical arrangements of parents and children are combined to form a tree. “Virtual scaffolds” that do not exist in the dataset are constructed *in silico*. Each node in the tree denotes a scaffold. A parent scaffold is a substructure of a child scaffold, and while every child scaffold only links to one parent in the scaffold tree, a parent scaffold can be the common substructure shared between many different children scaffolds. The children scaffolds that share the same parent scaffold are termed “sibling scaffolds”. It is worth noting that each compound can only be assigned to one scaffold node. For a compound belonging to a specific scaffold node, it signifies that the largest core structure of this compound matches exactly or is identical with the scaffold structure assigned at this node.

In this work, we applied Scaffold Hunter to construct scaffold trees in order to illustrate the relationships between mutagenic and non-mutagenic scaffolds. These hierarchical trees assist with the visual analyses of parent-child and sibling structural relationships.

### Cutoffs for Selecting Mutagenic and Non-Mutagenic Scaffolds

We assigned a mutagenicity value to each scaffold for reorganization of representative mutagenic and non-mutagenic scaffolds in the scaffold tree. The mutagenicity of a scaffold was defined as the ratio of mutagenic to total compounds categorized in that scaffold. A mutagenicity cutoff was then specified for selecting representative mutagenic and non-mutagenic scaffolds. The scaffolds whose mutagenicities are greater than or equal to the mutagenicity cutoff are defined as representative mutagenic scaffolds whereas the scaffolds whose mutagenicities are less than the mutagenicity cutoff are defined as representative non-mutagenic scaffolds. In addition, mutagenic and non-mutagenic scaffolds have to cover at least 10 compounds.

The mutagenicity cutoff was adjusted to select a minimal number of mutagenic scaffolds covering a maximum number of mutagenic compounds (mutagens). This means selecting a minimal set of scaffolds that represented as many mutagens as possible. Thus, we maximized the ratio (C_1_/S) of the number of mutagens (C_1_) to the number of mutagenic scaffolds (S) when adjusting the cutoff obtained using the selection criteria above. The detailed steps for selection of best mutagenicity cutoff were demonstrated in results and discussion.

Additionally, the non-mutagenicity cutoff was adjusted to select a minimum number of non-mutagenic scaffolds covering the maximum number of non-mutagenic compounds (non-mutagens). Therefore, we sought to select the minimum set of scaffolds that could represent as many non-mutagens as possible. Accordingly, we maximized the ratio (C_2_/S) of the number of non-mutagens (C_2_) to the number of non-mutagenic scaffolds (S) selected using the given cutoff criteria.

The adjustment of mutagenicity cutoffs and the selection criteria for choosing representative mutagenic and non-mutagenic scaffolds, are discussed in length under the “Selection Criteria for Mutagenic and Non-Mutagenic Scaffolds” section below.

## Results and Discussion

**Fig 1:**
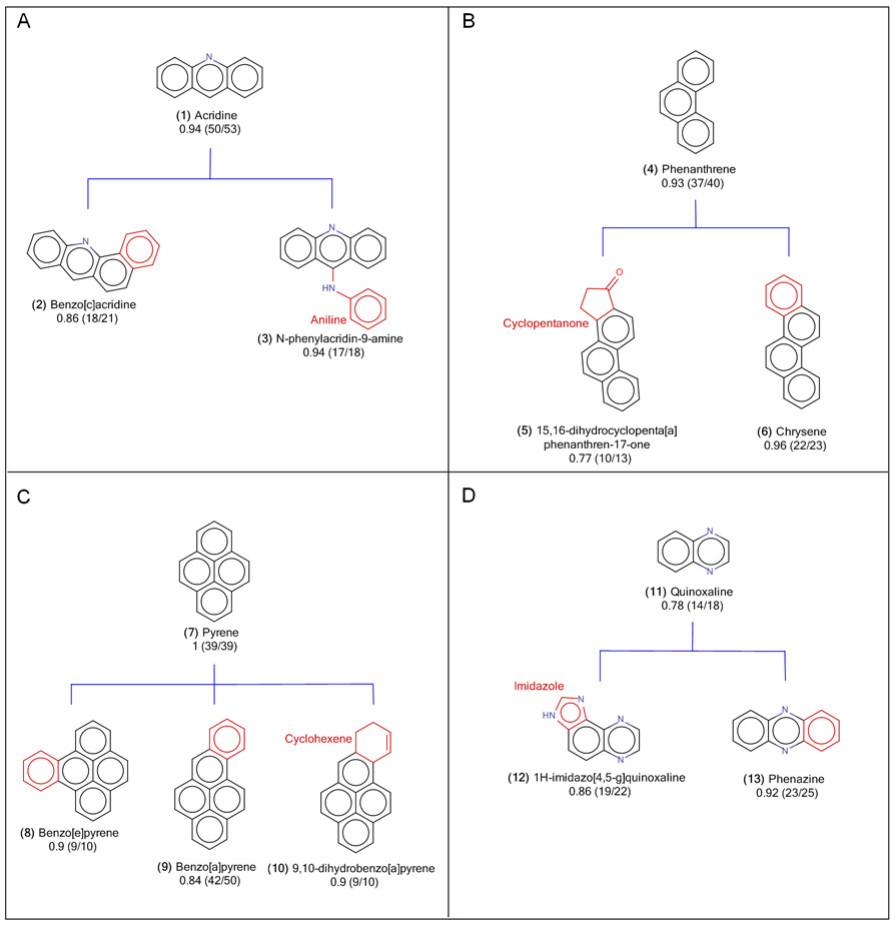
The scaffold structures of major mutagenic scaffold groups. (A) Acridine, (B) Phenanthene, (C) Pyrene, and (D) Quinoxaline groups. Below the labels of scaffold names were labelled by the mutagen rates and the numbers of mutagen compounds/the numbers of overall compounds in that scaffolds. In the structures of child scaffolds, the differences from the parent scaffold were colored as red. The mutagenicities shown in Fig 1 were presented as the percentage of mutagenic compounds for each scaffold, and the IUPAC names were generated using the Chemaxon Marvin applet.[30].

In this section, we will first describe and discuss the selection criteria for choosing representative mutagenic and non-mutagenic scaffolds. Then, we will discuss scaffold-mutagenicity relationship between major and minor mutagenic scaffolds and their “children” scaffolds. In this study, a scaffold is defined as a fixed part of a molecule, on which functional groups or other side chains can be substituted or exchanged. A mutagenic scaffold is defined as the scaffold that meets the following specifications: (1) a scaffold with mutagenicity (score) greater than the pre-determined selection criteria, and (2) there should be at least ten compounds with this scaffold as part of their structures. The “children” scaffolds in this study, refer to the variation of molecules belonging to a family of molecules sharing an identical (fixed) scaffold. **Fig 1**provides an illustration of the hierarchy and organization between four mutagenic parent scaffolds and children scaffolds.

When all of the children of a mutagenic scaffold are also mutagenic, we defined those children scaffolds and their parent mutagenic scaffold as a group of “major mutagenic scaffolds”. These children scaffolds are considered mutagenic if there are at least ten compounds with these children scaffolds present as part of their structures in the Ames dataset. On the other hand, for the scaffolds with mutagenicity (score) lower than but close to the selection criteria, we defined those children and their parent scaffold as a group of “minor mutagenic scaffolds”. Different selection criteria were applied to successfully identify scaffolds containing the most mutagenic compounds. If a series of compounds including a scaffold and its children scaffolds are all mutagenic, then we may infer that those scaffolds contribute significantly to mutagenicity. Therefore, all scaffolds satisfying the selection criteria were discussed according to their scaffold structures and substructures (children scaffolds). Finally, we further recognized the reduction rules to elucidate how to modify a mutagenic compound into a non-mutagenic molecule. In order to specify these rules, each group of the selected scaffolds was then compared with the substructure between parent and child scaffolds.

### Selection Criteria for Mutagenic and Non-Mutagenic Scaffolds

**Fig 2:**
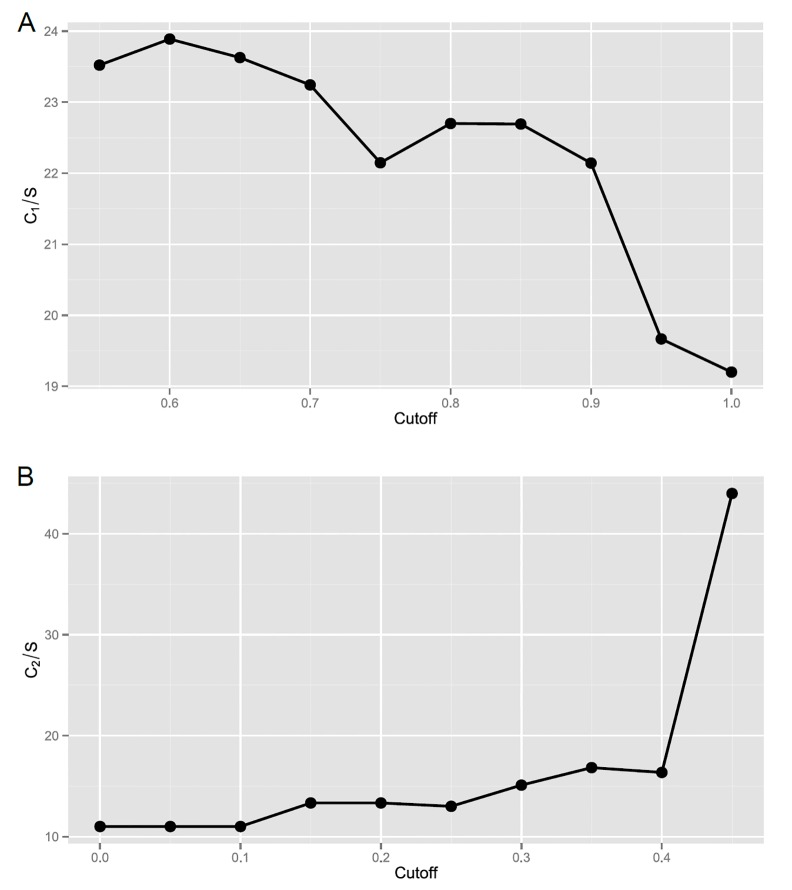
The C/S distribution plots according to different mutagenicity cutoff values. (A) In selecting mutagenic scaffolds, using the mutagens categorized in each selected scaffold as the selection criteria (C_1_/S). The detailed scores were listed in **Table A in S1 File**. (B) In selecting non-mutagenic scaffolds, using the non-mutagens categorized in each selected scaffold as the selection criteria (C_2_/S). The detailed scores were listed in **Table B in S1 File.** (C_1_: number of mutagenic compounds, C_2_: number of non-mutagenic compounds, S: number of mutagenic (for C_1_) or non-mutagenic (for C_2_) scaffolds).

For selection of mutagenic scaffolds, ten different mutagenicity cutoff percentages (100%, 95%, 90%, 85%, 80%, 75%, 70%, 65%, 60% and 55%) were applied initially, to search for an appropriate cutoff point in order to optimally differentiate the significance of mutagenicity between all of the core structures in our established scaffold tree. In doing so, we aimed to yield a minimum number of scaffolds covering the maximum number of mutagens. The C_1_/S distribution was plotted against ten different mutagenicity cutoff percentages in **Fig 2A**, where S represents the number of scaffolds selected, and C_1_ represents the number of mutagens categorized into the selected scaffolds. The detailed values of the C_1_/S distribution plot were listed in **Table A in S1 File**. The ratios of C_1_ to S resulting in 100%, 95%, 90%, 85%, 80%, 75%, 70%, 65%, 60% and 55% mutagenicity were 19.2 (96/5, showing total of 96 mutagens and 5 selected scaffolds), 19.7 (118/6), 22.1 (310/14), 22.7 (590/26), 22.7 (681/30), 22.1 (753/34), 23.2 (860/37), 23.6 (1016/43), 23.9 (1075/45), and 23.5 (1082/46), respectively. Although 60% (0.6) mutagenicity cutoff resulted in the highest ratio of C_1_, mutagens number categorized in the selected scaffolds, to S, selected scaffolds number, 60% mutagenicity cutoff isn’t statistically meaningful. We then selected a higher mutagenicity cutoff that still retains high value of C_1_/S. Although the difference between C_1_/S ratios of 0.6 and 0.7 isn’t significant (**Table A in S1 File**), the selection of mutagenicity cutoff that is higher than 0.7 will result in a loss of more than 20% mutagenic compounds. Therefore, 70% (0.7) mutagenicity cutoff point was chosen for evaluation of Ames mutagenicity.

For selection of non-mutagenic scaffolds, ten mutagenicity cutoff percentages (45, 40, 35, 30, 25, 20, 15, 10, 5 and 0%) were chosen, to select a minimum number of scaffolds covering the maximum number of non-mutagens. The C_2_/S distribution was plotted against the ten different mutagenicity cutoff values shown above in **Fig 2B**, and detailed values were listed in **Table B in S1 File**. Similar to the definitions above, S represents the number of scaffolds selected, and C_2_ represents the number of non-mutagens categorized as the selected scaffolds. The ratios of C_2_ to S resulting in 45, 40, 35, 30, 25, 20, 15, 10, 5 and 0% mutagenicity were 44 (924/21), 16.4 (229/14), 16.8 (202/12), 15.1 (151/10), 13 (104/8), 13.3 (80/6), 13.3 (80/6), 11 (22/2), 11 (22/2), and 11 (22/2), respectively. Similar to the rationale for selecting the best adjusted C_1_/S cutoff, although 45% (0.45) mutagenicity cutoff yielded the highest ratio of C_2_ to S, we opted for a lower mutagenicity cutoff percentage that still retains low value of C_2_/S. Since the selection of mutagenicity cutoff of less than 0.3 will result in loss of more than 80% of non-mutagenic compounds covered by scaffolds selected, 35% (0.35) mutagenicity was then chosen. For simplicity, the mutagenicity cutoff points will be referred to as mutagenicity scores for the purpose of this discussion.

In general, the ratios of C_1_ to S were higher than the ratios of C_2_ to S, this indicates that mutagens share more common scaffolds and chemical attributes compare to non-mutagens. Thus, this study focuses on the mutagens to identify these common scaffolds which contribute toward mutagenicity. Finally, after the appropriate selection criteria were determined, 37 mutagenic scaffolds (with mutagenicity score ≧ 0.7) and 12 non-mutagenic scaffolds (with mutagenicity score ≦ 0.35) were identified and summarized in **Table D in S1 File**.

### Major Mutagenic Scaffolds from the Ames Mutagenicity Scaffold Tree

A total of 6512 compounds were included in our dataset. To organize the scaffolds covered in this dataset for easier interpretations, a scaffold tree was generated using Scaffold Hunter. This scaffold tree comprises 12 layers with total of 2456 scaffolds. On average, each scaffold covered 4 compounds. From the assessment of Ames mutagenicity, 49 out of 2456 scaffolds were recognized as representative scaffolds present in more than 10 compounds, with mutagenicity scores ≧ 0.7 or ≦ 0.35. Of these representative scaffolds, 37 scaffolds have mutagenicity scores ≧ 0.7, and 12 scaffolds have mutagenicity scores ≦ 0.35. In another word, 37 scaffolds were identified from 996 compounds, and at least 70% of these compounds were known to be mutagenic (860 tested Ames positive and 136 Ames negative compounds). Similarly, 12 scaffolds were identified from 259 compounds and only less than 35% of these compounds were known to be mutagenic (57 Ames positive and 202 Ames negative compounds). Those scaffolds resulting in more than 70% of the compounds being mutagenic, were strongly correlated with mutagenicity. In contrast, those scaffolds resulting in less than 35% of the compounds being mutagenic, were considered to have low tendency towards mutagenicity (non-mutagenic).

To determine the common structural features (or major mutagenic scaffolds) that contribute significantly toward mutagenicity, structural relationship between the 49 scaffolds were examined. Any scaffolds sharing a common structural feature were grouped together. Thus, a scaffold tree was built for a group of scaffolds sharing a common structural feature, and their structural relationship was depicted as direct parent and child correlation. From the 37 mutagenic scaffolds identified above, 13 scaffolds shared structural similarities, and they were categorized into four groups as shown in **Fig 1**. The four major mutagenic scaffolds are acridine **(1)**, phenanthrene **(4)**, pyrene **(7)**, and quinoxaline **(11)** groups. All of the children scaffolds listed under the four parent scaffolds have mutagenicity scores ≧ 0.7, and every children scaffold was present in at least 10 compounds as their core structures (**Fig 1**). The statistics concerning the rate of mutagens found and the number of compounds for each scaffold under the four major mutagenic groups were listed in the first four rows of **Table C in S1 File**. The analysis reported here demonstrated that compounds bearing any one of these four major mutagenic scaffolds are very likely to induce mutagenicity regardless of their side chain modifications. The structural characteristics of the major mutagenic scaffolds are discussed in the following sections.

### Major Mutagenic Scaffolds (I): Acridine Group

In the benchmark Ames mutagenicity dataset, acridine was considered one of the major mutagenic scaffolds because more than 70% of the compounds (94%, 50/53 compounds) with acridine **(1)** as core structure were mutagenic. For example, N-acridin-9-yl-N',N'-dimethylpropane-1,3-diamine and 2-[[9-[3-(dimethylamino)propylamino]-1-nitroacridin-4-yl]-(2-hydroxyethyl)amino]ethanol both contain acridine in their structures, and they were both tested positive in Ames test, significantly induced colony growth in at least one out of five Salmonella strains. The acridine scaffold tree consists of six children scaffolds, however, four of the six children scaffolds did not meet the selection criteria. These four scaffolds (not shown) were found in 7 (< 10) compounds in the benchmark dataset, and only 3 out of the 7 compounds were tested positive for Ames mutagenicity, suggesting that these four children scaffolds do not contribute significantly toward mutagenicity. For this reason they were excluded from the acridine scaffold tree and discussion.

The two children scaffolds that were shown in the parent-children scaffold tree included benzo[c]acridine **(2)** and N-phenylacridin-9-amine **(3)** (**Fig 1**). They were considered major mutagenic scaffolds because the mutagenicity score of the two children scaffolds are higher than 0.7. While 94% of acridine-containing compounds are mutagenic (from the total of 53 compounds), not all of the benzo[c]acridine **(2)** and N-phenylacridin-9-amine **(3)** containing compounds are mutagenic. From the total of twenty-one benzo[c]acridine **(2)** compounds, 86% were mutagens, and from the total of eighteen N-phenylacridin-9-amine **(3)** compounds, 94% were mutagenic (**Table C in S1 File**).

The differences between the children acridine **(1)** scaffolds and the parent acridine **(1)** scaffold are: one of the children scaffold, benzo[c]acridine **(2)**, has an additional benzene ring compared to the parent scaffold, and the other children scaffold, N-phenylacridin-9-amine **(3)**, has an aniline structure added to the parent scaffold. Benzo[c]acridine contains an additional benzene substructure compared to acridine **(1)**, and we observed the mutagenicity of benzo[c]acridine **(2)** was thus slightly decreased (**Table C in S1 File**). An example of a benzo[c]acridine **(2)** containing compound is 7,11-dimethylbenzo[c]acridine. This compound has two methyl groups added to benzo[c]acridine, and it was tested positive for mutagenicity according to Ames test.

The structure of N-phenylacridin-9-amine **(3)** has an aniline group added to the dihydropyridine of acridine **(1)**, yet the mutagenicity of N-phenylacridin-9-amine **(3)** is similar to that of acridine **(1)** parent scaffold (**Table C in S1 File**). Amsacrine is a drug clinically used in the treatment of acute leukaemia. Its structure composed of a methoxy group and a methylsulfonyl group attached to the N-phenylacridin-9-amine **(3)** core structure, and it has been investigated extensively for its well-known mutagenicities.[31] These examples demonstrated that compounds containing acridine **(1)** scaffold have a higher tendency of being mutagenic, but also structural modifications on the acridine **(1)** scaffold resulting in children scaffolds such as benzo[c]acridine **(2)** and N-phenylacridin-9-amine **(3)**, still preserves the high mutagenicity tendency of acridine **(1)**. Therefore, from the evidence presented above, acridine **(1)**, benzo[c]acridine **(2)**, and N-phenylacridin-9-amine **(3)** scaffolds were collectively classified into one major mutagenic group.

### Major Mutagenic Scaffolds (II): Phenanthrene Group

Phenanthrene **(4)** was considered the second major mutagenic scaffold from our analysis of benchmark Ames dataset because 93% of the phenanthrene-containing compounds (from a total of 40 compounds) were mutagens. Phenanthren-1-amine is an example of a phenanthrene **(4)** scaffold containing compound from the Ames mutagenicity benchmark dataset, with positive Ames test result. The phenanthrene **(4)** parent scaffold consists of two mutagenic children scaffolds (**Fig 1B**), and eleven non-mutagenic children scaffolds (not shown). Although most of the phenanthrene **(4)** children scaffolds were non-mutagenic, each of the non-mutagenic scaffold was only present in an average of 2 compounds, while the two mutagenic children scaffolds covered more than 10 compounds each. Furthermore, the mutagenicity scores reported for half of the non-mutagenic children scaffolds were very low (< 0.5). Therefore, we can reasonably ignore the phenanthrene **(4)** children scaffolds that do not contribute significantly to mutagenicity.

The structural relationship between the two mutagenic children scaffolds and phenanthrene **(4)** was shown in **Fig 1B**. The first of the two children scaffolds, 15,16-dihydrocyclopenta[a]-phenanthren-17-one **(5)**, was present in 13 compounds, and 77% of these compounds were known to be mutagenic. We can observe that 15,16-dihydrocyclopenta-[a]phenanthren-17-one **(5)** has an added cyclopentanone substructure compared to the phenanthrene **(4)** parent scaffold. Interestingly, this addition led to a 16% decrease in mutagenicity of 15,16-dihydrocyclopenta-[a]phenanthren-17-one **(5)** compared to phenanthrene **(4)** (**Table C in S1 File**). Examples of compounds containing 15,16-dihydrocyclopenta-[a]phenanthren-17-one **(5)** scaffold are "11,12-dimethyl-15,16-dihydrocyclopenta[a]phenanthren-17-one", and "16-hydroxy-11-methyl-15,16-dihydrocyclopenta[a]phenanthren-17-one". These two compounds contain an additional methyl, and an hydroxyl group attached on the phenanthrene **(4)** and cyclopentanone of 15,16-dihydrocyclopenta-[a]phenanthren-17-one **(5)** scaffold, respectively.

The other mutagenic children scaffold, chrysene **(6)**, was responsible for 96% of mutagenicity from the total of 23 compounds with chrysene as their core structure. This indicates that regardless of the addition of benzene to the phenanthrene **(4)** parent scaffold, the high mutagenicity rate found in phenanthrene-containing compounds was reflected in chrysene-containing compounds. An example of a mutagenic compound with chrysene **(6)** as its core structure is 2-nitrochrysene. This section demonstrated that phenanthrene **(4)**, 15,16-dihydrocyclopenta[a]phenanthren-17-one **(5)** and chrysene **(6)** have direct parent-children scaffolds structural relationship, but also all of these scaffolds contribute significantly toward compound mutagenicity. Hence, these scaffolds were organized into one major mutagenic group.

### Major Mutagenic Scaffolds (III): Pyrene Group

Pyrene **(7)** was the third major mutagenic scaffold, but it may be the most important out of the four major mutagenic scaffolds identified in this study, because all of the compounds (39 total) with pyrene **(7)** core structure were mutagenic. This suggests that a pyrene-containing compound usually has the potential to induce mutagenicity. For this reason, any compound with pyrene **(7)** scaffold as part of its structure should be carefully avoided in the drug candidate selection processes. It shouldn’t be surprising that pyrene **(7)** itself was proven to be mutagenic according to the test performed in different Salmonella strains, including TA97, TA98, TA100 and TA1537 when S9 was present.[32] 1,8-dinitropyrene, and N-(6-hydroxypyren-1-yl)acetamide are two examples of mutagens containing pyrene **(7)** scaffold as part of their structures.

The pyrene **(7)** scaffold tree consists of thirteen children scaffolds, including three mutagenic children scaffolds and ten non-mutagenic children scaffolds. Although ten of the pyrene **(7)** children scaffolds were non-mutagenic, each of the non-mutagenic scaffold was only presented in an average of 3 compounds, while the three mutagenic children scaffolds were represented in at least 10 compounds each, with high mutagenicity (rate of mutagen) as shown in **Table C in S1 File**. The three mutagenic children scaffolds are benzo[e]pyrene **(8)**, benzo[a]pyrene **(9)** and 9,10-dihydrobenzo[a]pyrene **(10) (Fig 1C)**. The benzo[e]pyrene **(8)** and benzo[a]pyrene **(9)** scaffolds yielded the overall mutagenicity rate of 90% from a total of 10 benzo[e]pyrene-containing compounds, and 84% from a total of 50 benzo[a]pyrene-containing compounds, respectively (**Table C in S1 File**).

Both benzo[e]pyrene **(8)** and benzo[a]pyrene **(9)** structures have an additional benzene ring attached to the pyrene **(7)** scaffold, and the letters [e] and [a] denotes the location to which ring-fusion occurred. The additional benzene ring in benzo[e]pyrene **(8)** decreased mutagenicity by 10%, while the additional benzene ring in benzo[a]pyrene **(9)** decreased mutagenicity by 16%, compared to the pyrene **(7)** parent scaffold (100% mutagenicity; **Table C in S1 File**). This demonstrated that attachment of the same chemical substituent on different locations of a core structure could change the chemical properties of a scaffold, making it more or less mutagenic. Examples of two mutagens containing benzo-[e]pyrene **(8)** and benzo[a]pyrene **(9)** scaffold respectively, are 1-nitrobenzo[l]pyren-8-ol and 5-(chloromethyl)benzo[a]pyrene, according to the Ames test.

The third child scaffold, 9,10-dihydrobenzo[a]pyrene **(10)**, contains a cyclohexene ring attached to pyrene **(7)**. This addition yielded the overall mutagenicity of 90% from a total of 10 selected compounds (**Table C in S1 File**). An example of a known mutagen with 9,10-dihydrobenzo[a]pyrene **(10)** core structure is (7S,8S)-3-nitro-7,8-dihydrobenzo[a]pyrene-7,8-diol. It was suggested that pyrene **(7)**, benzo[e]pyrene **(8)**, benzo[a]pyrene **(9)** and 9,10-dihydrobenzo[a]pyrene **(10)** were four noteworthy scaffolds that could cause mutagenicity and were classified in our third major mutagenic group.

### Major Mutagenic Scaffolds (IV): Quinoxaline Group

Quinoxaline **(11)** was considered the fourth major mutagenic scaffold in this study, with the overall mutagenicity rate of 78% from a list of 18 quinoxaline **(11)** compounds identified from Ames dataset Two examples of mutagenic compounds containing quinoxaline **(11)** scaffold as part of their structures, are 2,3-dimethoxy-5-methylquinoxaline and 5-(bromomethyl)-2,3-dimethoxy-7-nitroquinoxaline. There were four quinoxaline **(11)** children scaffolds identified, they are: “1H-imidazo[4,5-g]quinoxaline” **(12)**, “N-(quinoxalin-2-yl)benzenesulfonamide”, “2-phenylquinoxaline”, and phenazine **(13)**. "N-(quinoxalin-2-yl)benzenesulfonamide", and "2-phenylquinoxaline" were children scaffolds that did not contribute significantly toward mutagenicity since they were reported to be 0% and 50% mutagenic. The last two children scaffolds that were classified as major mutagenic scaffolds in quinoxaline **(11)** group include 1H-imidazo[4,5-g]quinoxaline **(12)** and phenazine **(13)** (**Fig 1D**). 1H-imidazo[4,5-g]quinoxaline **(12)** contains an additional structure of imidazole on the benzene of quinoxaline **(11)**, and covered 19 mutagens out of 22 compounds, while phenazine **(13)** includes a structure of benzene attached on quinoxaline **(11)**, and covered 23 mutagens out of 25 compounds. The core structures modified by the imidazole and benzene on the structure of quinoxaline **(11)**all resulted in higher mutagenicity than quinoxaline **(11)** itself (**Table C in S1 File**). Examples of compounds containing structures of 1H-imidazo[4,5-g]quinoxaline **(12)** and phenazine **(13)** are 3,4,8-trimethylimidazo[4,5-f]quinoxalin-2-amine and 1,7-dinitrophenazine, respectively. Since both 1H-imidazo[4,5-g]quinoxaline **(12)** and phenazine **(13)** are mutagenic children scaffolds of the quinoxaline **(11)** parent scaffold, and the two children scaffolds have higher potential to cause mutagenicity compared to the parent scaffold, it would be favorable to not include these in the core structures of drug candidates.

### Minor Mutagenic Scaffolds of the Ames Mutagenicity Tree

**Fig 3:**
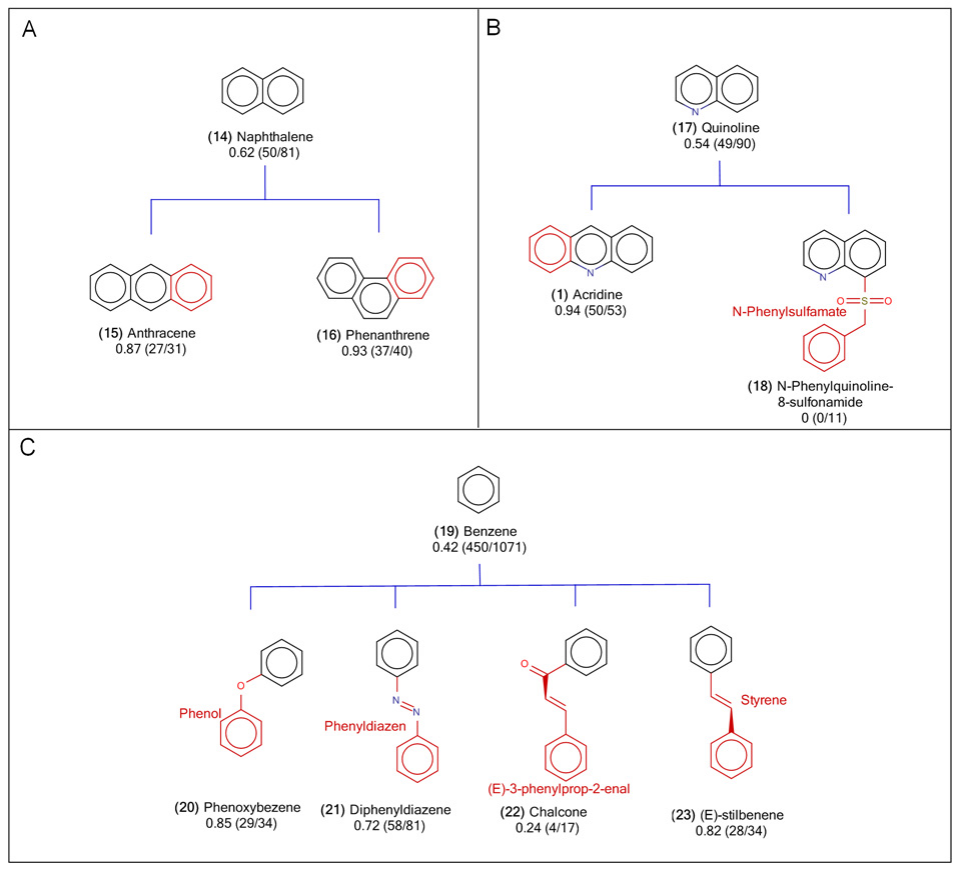
The scaffold structures of minor mutagenic scaffold groups. (A) Naphthalene, (B) Quinoline, and (C) Bezene groups. Below the labels of scaffold names were labelled by the mutagen rates and the numbers of mutagen compounds/the numbers of overall compounds in that scaffolds. In the structures of child scaffolds, the differences from the parent scaffold were colored as red.

Naphthalene group consists of minor mutagenic scaffolds, since the mutagenicity scores for the scaffolds selected for this group are between 0.35 and 0.7. As shown in the fifth row of **Table C in S1 File**, 62% of the compounds with naphthalene **(14)** scaffold as part of their structures are mutagenic. Examples of mutagenic naphthalene-containing **(14)** compounds include 1-(4-methoxynaphthalen-1-yl)prop-2-enyl acetate and N-hydroxy-N-naphthalen-2-ylformamide. In the naphthalene **(14)** group, two children scaffolds, anthracene **(15)** and phenanthrene **(16)**, were identified and their structures were shown in **Fig 3A**. In both children scaffolds, the fusion of naphthalene **(14)** with an additional benzene ring at different locations on naphthalene **(14)**, resulted in much higher mutagenicity overall compared to that of naphthalene **(14)** itself. In anthracene **(15)**, the addition of benzene on naphthalene **(14)** yielded 87% mutagens from a total of 31 anthracene-containing **(15)** compounds, while in phenanthrene **(16)**, the addition of benzene on naphthalene **(14)** at a different fusion location resulted in 93% mutagens from a total of 40 phenanthrene-containing **(16)** compounds (**Table C in S1 File**). The following compounds, 3-methylanthracene-1,8,9-triol and 2,10-dinitrophenanthrene, are known mutagens containing anthracene **(15)** and phenanthrene **(16)** children scaffolds, respectively. Although naphthalene **(14)** parent scaffold does not contribute significantly toward causing mutagenicity, both anthracene **(15)** and phenanthrene **(16)** children scaffolds are linked to higher rate of mutagens, for this reason it was deduced that compounds with naphthalene **(14)** group should serve as warning scaffolds for mutagens.

In addition to the naphthalene **(14)** group, the children scaffolds of benzene **(19)** (**Fig 3C)** and quinoline **(17)** (**Fig 3B**) groups covered 3 of the 37 recognized mutagenic scaffolds. However, these two groups were not considered as minor mutagenic scaffolds. When scaffolds that are present in less than 10 compounds were removed, three out of ten children scaffolds under benzene **(19)** group yielded mutagenicity score greater than 0.7, and one out of ten scaffolds produced mutagenicity less than 0.35. The four structures mentioned above included phenoxybenzene **(20)**, diphenyldiazene **(21)**, chalcone **(22)** and (E)-stilbene **(23)** (**Fig 3C**). Benzene **(19)** contained 42% mutagens (out of 1071 compounds), phenoxybenzene **(20)** contained 85% mutagens (out of 34), diphenyldiazene **(21)** had 72% (out of 81), chalcone **(22)** had 24% (out of 17) and (E)-stilbene **(23)** had 82% (out of 34) (**Table C in S1 File**). However, since the benzene **(19)** scaffold is a common structure that covers most compounds with a broad range of mutagenicity, benzene **(19)** cannot be regarded as a mutagenic scaffold group. Quinoline **(17)** was the third scaffold with mutagenicity score between 0.3 and 0.7 (54% mutagens, out of 90 compounds). However, quinoline **(17)** was not recognized as a minor mutagenic scaffold either, because only one of the scaffolds in the quinolone **(17)** group was mutagenic. The quinoline **(17)** group contains two children scaffolds, N-phenylquinoline-8-sulfonamide **(18)** and N-phenylsulfamate, each covering more than 10 compounds at least from the Ames dataset. As shown in **Fig 3B**, N-phenylquinoline-8-sulfonamide **(18)** is one of the children scaffolds, which was classified as a mutagenic scaffold containing 94% mutagens (out of 53 compounds) while N-phenylsulfamate was the non-mutagenic scaffold with no mutagens (out of 11 compounds) **(Table C in S1 File)**.

### Reduction of Mutagenicity via Substructure Modification on Scaffolds

**Fig 4:**
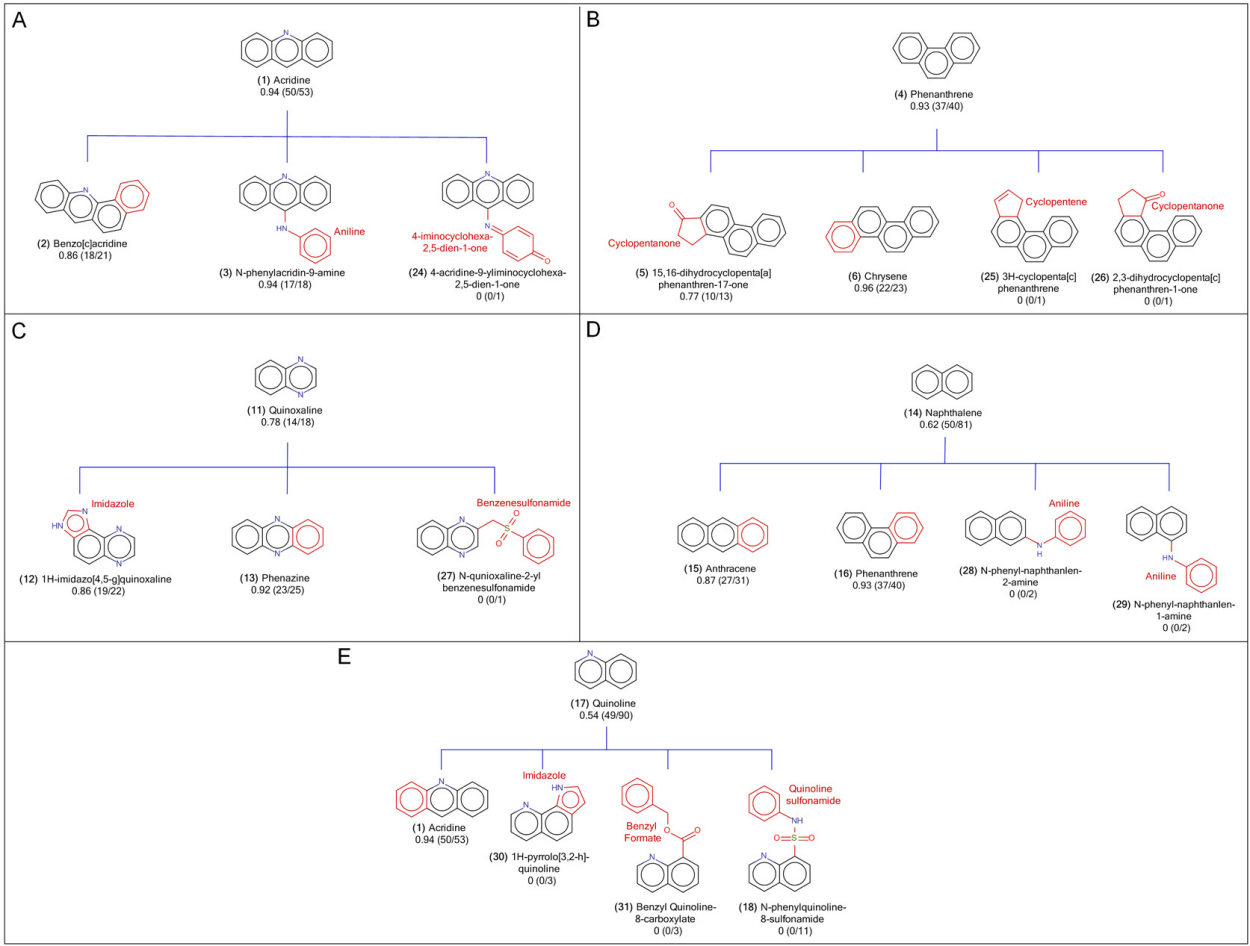
The scaffold structures of examples of sibling relationships between mutagenic scaffolds and non-mutagenic scaffolds. (A) Acridine, (B) Phenanthrene, (C) Quinoxaline, (D) Naphthalene, and (E) Quinoline groups. Below the labels of scaffold names were labelled by the mutagen rates and the numbers of mutagen compounds/the numbers of overall compounds in that scaffolds. In the structures of child scaffolds, the differences from the parent scaffold were colored as red.

Most importantly, we have recognized a series of substructures that can be used to modify the mutagenic scaffolds for decreasing the mutagenic activities. By observing the variations of mutagenicity between children of non-mutagenic scaffolds and of the scaffolds in our recognized major/minor mutagenic groups, we induced five series of the reduction rules that can decrease mutagenicity by modifying the substructures of mutagenic scaffolds. The structural relationships of the scaffolds in the five cases were illustrated in **Fig 4**.

The first case involved three child scaffolds in acridine **(1)** group: benzo[c]acridine **(2)**, N-phenylacridin-9-amine **(3)** and 4-acridin-9-yliminocyclohexa-2,5-dien-1-one **(24)**. The structural relationship between the major mutagenic scaffold of acridine **(1)** and its three children scaffolds was shown in **Fig 4A**. The mutagenicity of benzo[c]acridine **(2)**, N-phenylacridin-9-amine **(3)** and 4-acridin-9-yliminocyclohexa-2,5-dien-1-one **(24)** were 86, 94 and 0%, respectively. The compounds containing benzo[c]acridine **(2)** have 86% probability to induce mutagenic activities according to the Ames test of our benchmark dataset. When we removed benzene from benzo[c]acridine **(2)** in those mutagenic compounds and then link a structure of 4-iminocyclohexa-2,5-dien-1-one on another fusion location of acridine **(1)**, the mutagenicity of the modified compounds (which now contains 4-acridin-9-yliminocyclohexa-2,5-dien-1-one scaffold) will be totally dispelled. Similarly, we can decrease mutagenicity of N-phenylacridin-9-amine **(3)** by replacing the aniline of N-phenylacridin-9-amine **(3)** with the structure of 4-iminocyclohexa-2,5-dien-1-one **(24)**.

The second case contained four children scaffolds under the major mutagenic scaffold of phenanthrene **(4)**: 15,16-dihydrocyclopenta[a]phenanthren-17-one **(5)**, chrysene **(6)**, 3H-cyclo-penta[c]phenanthrene **(25)** and 2,3-dihydrocyclopenta[c]phenanthren-1-one **(26)** (mutagenicity scores of 77%, 96%, 0%, and 0%, respectively; **Fig 4B**). The mutagenicity of 15,16-dihydrocyclopenta[a]phenanthren-17-one **(5)** can be reduced from 77 to 0% by substituting cyclopentene for the cyclopentanone on phenanthrene **(4)** of 15,16-dihydrocyclopenta[a]phenanthren-17-one **(5)** or rotating cyclopentanone by 180 degrees. We can also remove the mutagenicity of chrysene **(6)** by replacing the benzene ring on the phenanthrene **(4)** of chrysene **(6)** with a cyclopentene or cyclopentanone.

Three children of the major mutagenic scaffold of quinoxaline **(11)** consisting of 1H-imidazo[4,5-g]quinoxaline **(12)**, phenazine **(13)** and N-quinoxalin-2-yl-benzenesulfonamide **(27)** (**Fig 4C**). 1H-imidazo[4,5-g]quinoxaline **(12)** and phenazine **(13)** yielded high mutagenicity scores of 86% and 92%. When the imidazole on the structure of 1H-imidazo[4,5-g]quinoxaline **(12)** and a benzene on the structure of phenazine **(13)** were replaced by linking a benzenesulfonamide, the resultant scaffold of N-quinoxalin-2-yl-benzenesulfonamide **(27)** was obtained with no mutagenicity.

The fourth case involved four children scaffolds under the minor mutagenic scaffold of naphthalene **(14)**, and they were composed of anthracene **(15)**, phenanthrene **(16)**, N-phenylnaphthalen-2-amine **(28)** and N-phenylnaphthalen-1-amine **(29)** with mutagenicity scores of 87%, 93%, 0% and 0%, respectively (**Fig 4D**). To remove mutagenic activity of compounds that contain the structure of anthracene **(15)**, we can remove benzene from either site of anthracene **(15)**, and link an aniline on the resultant naphthalene **(14)**. In this way, the final compound contains the structure of N-phenylnaphthalen-2-amine **(28)** and yielded no mutagenic activity. Similarly, when we change the core structure of compounds containing phenanthrene **(16)** to the N-phenylnaphthalen-1-amine **(29)**, the mutagenicity of resultant compounds will be reduced.

The quinoline **(17)** group contains four children scaffolds: acridine **(1)**, 1H-pyrrolo[3,2-h]quinoline **(30)**, benzyl quinoline-8-carboxylate **(31)** and N-phenylquinoline-8-sulfonamide **(18)** (**Fig 4E**). The mutagenicity scores of these children scaffolds were 94%, 0%, 0%, and 0%, respectively. The mutagenicity of compounds which contained the scaffold of acridine **(1)** can be reduced from 94% to 0% by replacing the benzene ring with an imidazole, a benzyl formate or a quinolinesulfonamide on the benzene site of quinoline **(17)**.

### Comparison with the Structure Alert Approach (Toxtree)

**Fig 5:**
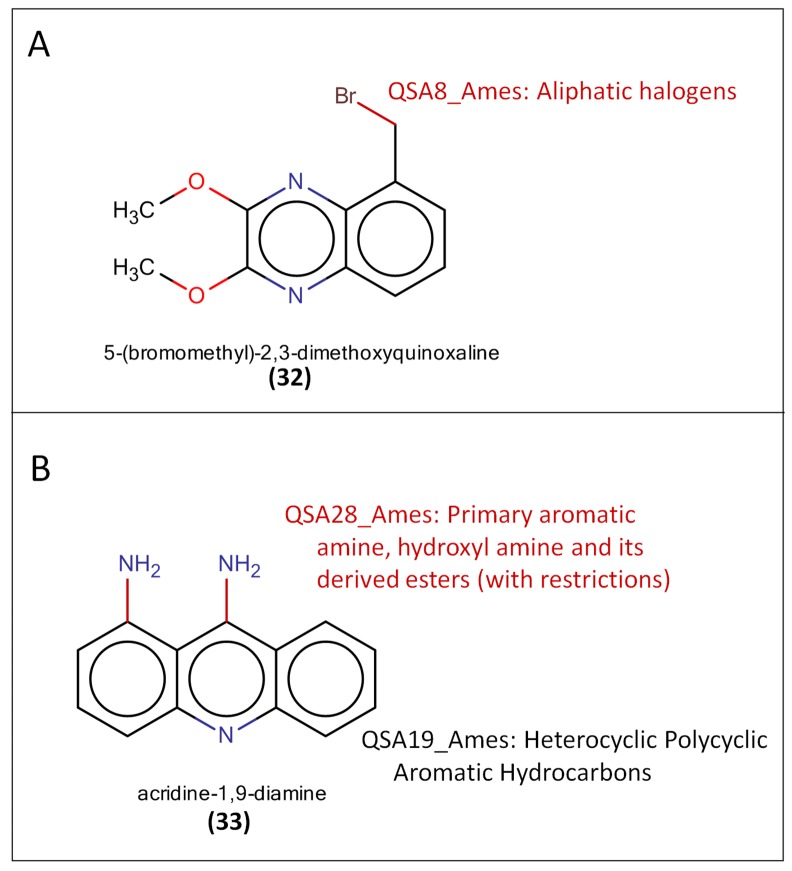
The Toxtree prediction results. (A) 5-(bromomethyl)-2,3-dimethoxyquinoxaline and (B) acridiine-1,9-diamine. The fitted structural alerts were labelled.

To cross check the mutagenicity analysis conducted in this study as well as the benefits of having scaffold-mutagenicity flags, the publicly available structure alert approach (Toxtree) was compared to the results of our analysis. All of the mutagens covered by our identified four major mutagenic scaffolds were tested by Toxtree. Mutagenic compounds covered by acridine, phenanthrene, and pyrene were all correctly predicted by our study and Toxtree. Two mutagens including 5-(bromomethyl)-2,3-dimethoxyquinoxaline **(32)** (quinoxaline scaffold), and acridine-1,9-diamine **(33)** (acridine scaffold) were taken as two examples successfully predicted by both our study and Toxtree. The two examples of predicted structural alerts for mutagenicity analyzed by Toxtree were presented in **Fig 5**. The matched structural alerts were highlighted and labeled in red text. According to the analysis of Toxtree, 5-(Bromomethyl)-2,3-dimethoxyquinoxaline **(32)** was predicted to be a mutagen due to the presence of an aliphatic halogen substructure alert. A similar result was observed for acridine-1,9-diamine **(33)**, which was predicted to be a mutagen in Toxtree due to a primary aromatic amine structure alert. Because the acridine scaffold is a major mutagenic scaffold, acridine-1,9-diamine **(33)** was also predicted to be a mutagen in this study.

However, quinoxaline **(11)** and 2,3-dimethoxy-5-methylquinoxaline containing the major mutagenic scaffold of quinoxaline **(11)** were the two mutagens predicted to be non-mutagenic in the analysis of Toxtree because no structure alerts were found. In our study, quinoxaline **(11)**, and 2,3-dimethoxy-5-methylquinoxaline were successfully predicted to be mutagens, as quinoxaline **(11)** is a major mutagenic scaffold.

Among the four major mutagenic scaffolds, the pyrene **(6)** groups with the mutagenicity score of 1 indicate that all pyrene-containing compounds are mutagens. All mutagenic compounds covered by pyrene were also correctly predicted by Toxtree since those compounds matched the structural alert, “SA_18: Polycyclic Aromatic Hydrocarbons (three or more fused rings)”. In fact, Toxtree contains very few scaffold-like structural alerts, such as “quinoes”, “halogenated benzene”, and “Polycyclic Aromatic Hydrocarbons”.[33] However, these general types of scaffold-like structural alerts could result in many false negatives. Take “Polycyclic Aromatic Hydrocarbons” as an example: many compounds having the properties of “Polycyclic Aromatic Hydrocarbons” (**Fig A in S1 File**) are actually non-mutagenic, such as fluoranthen-2-ylmethanol **(34)**, 1-{1-hydrazinyl-3H-pyridazino[4,5-b]indol-4-yl}-3,5-dimethyl-1H-pyrazole **(35)**, 9-nitro-1,2-dihydroanthracene-1,2-diol **(36)**, 9H-fluoren-9-one **(37)**, and 1-methyl-1H-imidazo[4,5-b]1,8-naphthyridin-2-amine **(38)**, etc. Therefore, the mutagenic scaffolds identified in our study can provide more structural specificity than the scaffold-like structural alerts in Toxtree. On the other hand, in our identified non-mutagenic scaffolds listed in **Table D in S1 File,** two non-mutagenic scaffolds with the mutagenicity of 0 including N-phenyl-quinoline-8-sulfonamide **(39)**, and 1,3,5-triazine **(40)**, indicate that all compounds containing the scaffold of **(39)** and **(40)** are non-mutagens. However, 59% of non-mutagens covered by **(39)** and **(40)** were predicted to be mutagenic in Toxtree, such as N-(3-nitrophenyl)quinoline-8-sulfonamide **(41)**, and 6-chloro-2-N,4-N-diethyl-1,3,5-triazine-2,4-diamine **(42)**, etc. (**Fig B in S1 File**) Those results demonstrated that the list of major mutagenic scaffolds, non-mutagenic scaffolds, and their Ames mutagenicity patterns recognized in our study can assist in predicting the core structures that are mutagenic, whereas Toxtree can only predict the substructures that are mutagenic. If the mutagenicity of a compound arises from its core structure instead of its substructural features, Toxtree will fail to identify such compound as a mutagen. Actually, the mutagenicities of most of our identified major or minor mutagenic scaffolds were less than 1. Therefore, the mutagenicity of compounds could depend on the some functional groups of chemical modification. Furthermore, partial of mutagens and non-mutagens still can be correctly predicted in Toxtree. We agreed that analysis of both functional group and scaffold of mutagenicity can enhance the predictability of mutagenicity of compounds.

## Conclusions

The major findings and conclusions of this study include: 1) all of the children scaffolds derived from major mutagenic scaffolds were also mutagenic; 2) parent scaffolds with insignificant mutagenicity may produce mutagenic or non-mutagenic children scaffolds depending on the attached substituent. Furthermore, 3) when the core scaffold rather than the side chains of a compound is responsible for the mutagenicity of that compound, modifications can be made by replacing the mutagenic core structure with a different structure to form non-mutagenic scaffolds. Detailed lists of major mutagenic scaffolds and suggestions for modifications of mutagenic scaffolds were provided.

The four major scaffolds contributing to Ames mutagenicity were acridine **(1)**, phenanthrene **(4)**, pyrene **(7)** and quinoxaline **(11)**. All of these scaffolds were mutagenic scaffolds containing at least 10 compounds, and the children scaffolds in each group were also mutagenic (**Fig C in S1 File**).

Furthermore, benzo[c]acridine **(2)** and N-phenylacridin-9-amine **(3)**, the child scaffolds of acridine **(1)**, also tended to be mutagenic. Likewise, 15,16-dihydrocyclopenta[a]phenanthren-17-one **(5)** and chrysene **(6)** (the child scaffolds of phenanthrene); benzo[e]pyrene **(8)**, benzo[a]pyrene **(9)** and 9,10-dihydrobenzo[a]pyrene **(10)** (the child scaffolds of pyrene); and 1H-imidazo[4,5-g]quinoxaline **(12)** and phenazine **(13)** (the child scaffolds of quinoxaline) also tended to be mutagenic (**Fig D in S1 File**).

In summary, acridine **(1)**, phenanthrene **(4)**, pyrene **(7)** and quinoxaline **(11)** were the four major scaffolds contributing to Ames mutagenicity according to the observation of the scaffolds, the grouping of the constructed scaffold tree, and the mutagenicity of each scaffold. Nine scaffolds (benzo[c]acridine **(2)**, N-phenylacridin-9-amine **(3)**, 15,16-dihydrocyclopenta[a]-phenanthren-17-one **(5)**, chrysene **(6)**, benzo[e]pyrene **(8)**, benzo[a]pyrene **(9)**, 9,10-dihydrobenzo-[a]pyrene **(10)**, 1H-imidazo[4,5-g]quinoxaline **(12)** and phenazine **(13)**) showed mutagenic tendencies. The lists of major scaffolds showing a tendency towards mutagenicity, and the suggested modifications of mutagenic scaffolds may be useful for drug development, especially during preclinical lead optimization and safety screening.

## Supporting Information

S1 File The additional Tables A-D, and Figures A-D. **Table A in S1 File**. The numbers of scaffolds selected, mutagens categorized as the selected scaffold and the ratios of C_1_ to S for different mutagenicity cutoffs. (C_1_: number of mutagenic compounds, S: number of mutagenic scaffolds); **Table B in S1 File**. The numbers of scaffolds selected, non-mutagens categorized as the selected scaffold and ratios of C_2_ to S for different mutagenicity cutoffs. (C_2_: number of non-mutagenic compounds, S: number of non-mutagenic scaffolds); **Table C in S1 File**. Rate of mutagen and number of compounds for each scaffold in: major mutagenic scaffold groups (Acridine, Phenanthrene, Pyrene, Quinoxaline), minor mutagenic scaffold group (Naphthalene), and scaffold groups which cannot be classified as either major or minor mutagenic groups (Benzene, Quinoline); **Table D in S1 File.** Overview of the selected scaffolds; **Fig A in S1 File.** The example of non-mutagenic compounds having the structural alert of “Polycyclic Aromatic Hydrocarbons”; **Fig B in S1 File.** The non-mutagenic compounds containing the non-mutagenic scaffolds of N-phenyl-quinoline-8-sulfonamide **(39)**, and 1,3,5-triazine **(40)**; **Fig C in S1 File.** The major scaffolds contributing to Ames mutagenicity; **Fig D in S1 File.** Scaffolds having mutagenic tendencies.
(PDF)
