# Supplementary material for: Mutagenicity in a Molecule: Identification of Core Structural Features of Mutagenicity Using a Scaffold Analysis
Source: PLoS One. 2016 Feb 10;11(2):e0148900. doi: 10.1371/journal.pone.0148900 (PMC4749256; doi:10.1371/journal.pone.0148900)
Supplement: S1 File — Table A in S1 File. The numbers of scaffolds selected, mutagens categorized as the selected scaffold and the ratios of C1 to S for different mutagenicity cutoffs. (C1: number of mutagenic compounds, S: number of mutagenic scaffolds); Table B in S1 File. The numbers of scaffolds selected, non-mutagens categorized as the selected scaffold and ratios of C2 to S for different mutagenicity cutoffs. (C2: number of non-mutagenic compounds, S: number of non-mutagenic scaffolds); Table C in S1 File. Rate of mutagen and number of compounds for each scaffold in: major mutagenic scaffold groups (Acridine, Phenanthrene, Pyrene, Quinoxaline), minor mutagenic scaffold group (Naphthalene), and scaffold groups which cannot be classified as either major or minor mutagenic groups (Benzene, Quinoline); Table D in S1 File. Overview of the selected scaffolds; Fig A in S1 File. The example of non-mutagenic compounds having the structural alert of “Polycyclic Aromatic Hydrocarbons”; Fig B in S1 File. The non-mutagenic compounds containing the non-mutagenic scaffolds of N-phenyl-quinoline-8-sulfonamide (39), and 1,3,5-triazine (40); Fig C in S1 File. The major scaffolds contributing to Ames mutagenicity; Fig D in S1 File. Scaffolds having mutagenic tendencies. (PDF) [file pone.0148900.s001.pdf]

Table A. The numbers of scaffolds selected, mutagens categorized as the selected scaffold and the ratios of C_1 to S for different mutagenicity cutoffs. **(C_1: number of mutagenic compounds, S: number of mutagenic scaffolds)**

**Mutagenicity Cutoff**	**Scaffolds Selected (S)**	**Mutagens Categorized as Selected Scaffold (C_1)**	**C_1/S**
1	5	96	19.2 (96/5)
0.95	6	118	19.7 (118/6)
0.9	14	310	22.1 (310/14)
0.85	26	590	22.7 (590/26)
0.8	30	681	22.7 (681/30)
0.75	34	753	22.1 (753/34)
0.7	37	860	23.2 (860/37)
0.65	43	1016	23.6 (1016/43)
0.6	45	1075	23.9 (1075/45)
0.55	46	1082	23.5 (1082/46)

Table B. The numbers of scaffolds selected, non-mutagens categorized as the selected scaffold and ratios of C_2 to S for different mutagenicity cutoffs. **(C_2: number of non-mutagenic compounds, S: number of non-mutagenic scaffolds)**

**Mutagenicity**	**Scaffolds Selected (S)**	**Non-Mutagens Categorized as Selected Scaffold (C_2)**	**C_2/S**
0.45	21	924	44 (924/21)
0.4	14	229	16.4 (229/14)
0.35	12	202	16.8 (202/12)
0.3	10	151	15.1 (151/10)
0.25	8	104	13 (104/8)
0.2	6	80	13.3 (80/6)
0.15	6	80	13.3 (80/6)
0.1	2	22	11 (22/2)
0.05	2	22	11 (22/2)
0	2	22	11 (22/2)

Table C. Rate of mutagen and number of compounds for each scaffold in: major mutagenic scaffold groups (Acridine, Phenanthrene, Pyrene, Quinoxaline), minor mutagenic scaffold group (Naphthalene), and scaffold groups which cannot be classified as either major or minor mutagenic groups (Benzene, Quinoline).

Scaffold Name	Rate of Mutagen	Compound Number
**Acridine Group**		
Acridine	94%	53
Benzo[c]acridine	86%	21
N-Phenylacridin-9-amine	94%	18
**Phenanthrene Group**		
Phenanthrene	93%	40
15,16-Dihydrocyclopenta[a]-phenanthren-17-one	77%	13
Chrysene	96%	23
**Pyrene Group**		
Pyrene	100%	39
Benzo[e]pyrene	90%	10
Benzo[a]pyrene	84%	50
9,10-Dihydrobenzo[a]pyrene	90%	10
**Quinoxaline Group**		
Quinoxaline	78%	18
1H-imidazo[4,5-g]quinoxaline	86%	22
Phenazine	92%	25
**Naphthalene Group**		
Naphthalene	62%	81
Anthracene	87%	31

Phenanthrene	93%	40
**Benzene Group**		
Benzene	42%	1071
Phenoxybenzene	85%	34
Diphenyldiazene	72%	81
Chalcone	24%	17
(E)-stilbene	82%	34
**Quinoline Group**		
Quinoline	54%	90
Acridine	94%	53
N-Phenylsulfamate	0%	11

Table D. Overview of the selected scaffolds.

Scaffold	Name	Positive Compounds /All Compounds	Ames Positive Rate
**Mutagenic Scaffolds**			
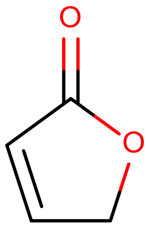	2(5H)-Furanone	23/25	0.92
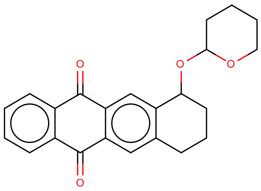	Aranciamycinone	14/14	1
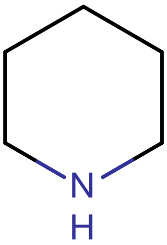	Piperidine	11/12	0.92
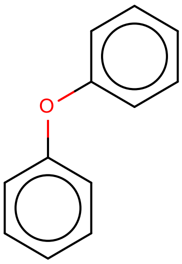	Phenoxybenzene	29/34	0.85
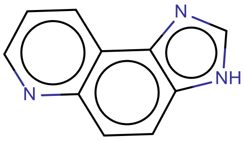	3H-imidazo[4,5-f]quinoline	13/14	0.93

Scaffold	Name	Positive Compounds /All Compounds	Ames Positive Rate
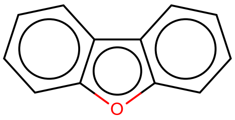	Dibenzofuran	9/12	0.75
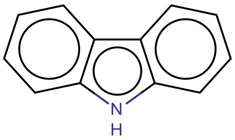	9H-carbazole	30/33	0.91
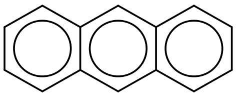	Anthracene	27/31	0.87
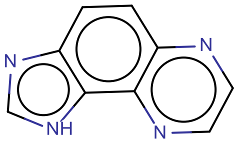	1H-imidazo[4,5-f]quinoxaline	19/22	0.86
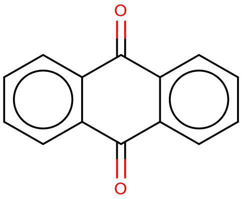	Anthracene-9,10-dione	59/69	0.86
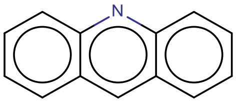	Acridine	50/53	0.94
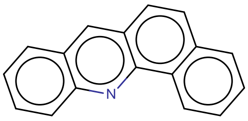	Benzo[c]acridine	18/21	0.86

**Scaffold**	**Name**	**Positive Compounds /All Compounds**	**Ames Positive Rate**
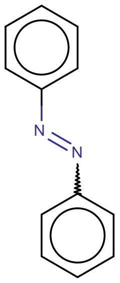	Diphenyldiazene	58/81	0.72
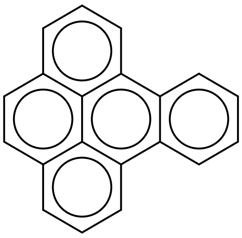	Benzo[e]pyrene	9/10	0.9
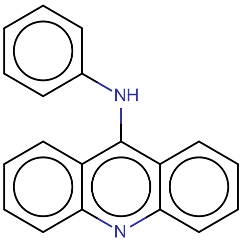	N-phenylacridin-9-amine	17/18	0.94
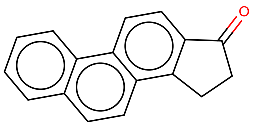	15,16-dihydrocyclopenta[a]phenanthren-17-one	10/13	0.77
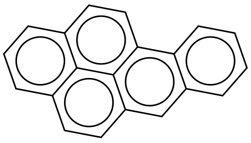	Benzo[a]pyrene	42/50	0.84
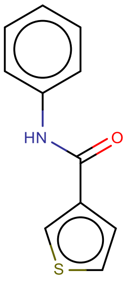	N-phenylthiophene-3-carboxamide	23/23	1

Scaffold	Name	Positive Compounds /All Compounds	Ames Positive Rate
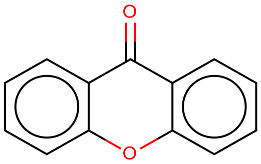	Xanthen-9-one	9/10	0.9
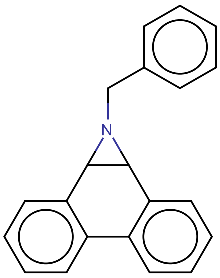	1-benzyl-1a,9b-dihydrophenanthro[9,10-b]azirine	10/10	1
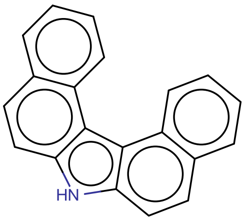	7H- dibenzo(C,G)carbazole	11/12	0.92
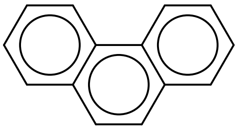	Phenanthrene	37/40	0.93
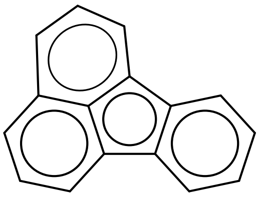	Fluoranthene	33/38	0.87
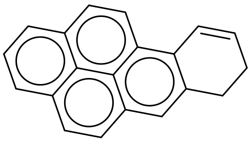	9,10-dihydro-benzo[a]pyrene	9/10	0.9

Scaffold	Name	Positive Compounds /All Compounds	Ames Positive Rate
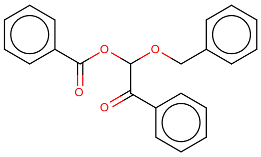	[benzoyl(phenylmethoxy)amino]benzoate	10/10	1
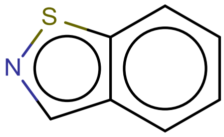	1,2-benzothiazole	15/17	0.88
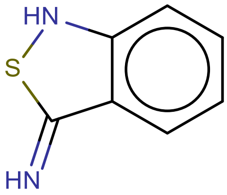	Benzisothiazoles	11/13	0.85
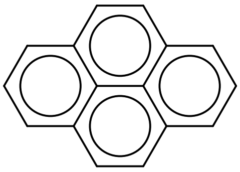	Pyrene	39/39	1
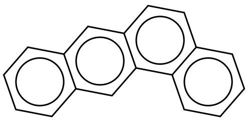	Benzo[a]anthracene	34/39	0.87
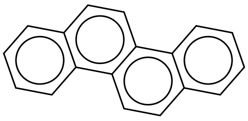	Chrysene	22/23	0.96
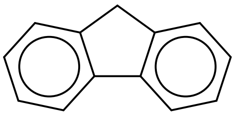	9H-fluorene	40/50	0.8
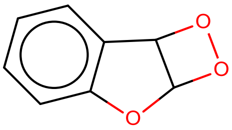	Benzofuran dioxetanes	10/12	0.83

**Scaffold**	**Name**	**Positive Compounds /All Compounds**	**Ames Positive Rate**
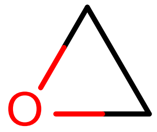	Oxirane	36/51	0.71
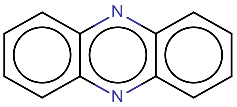	Phenazine	23/25	0.92
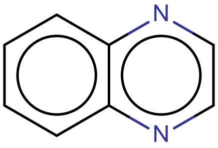	Quinoxaline	14/18	0.78
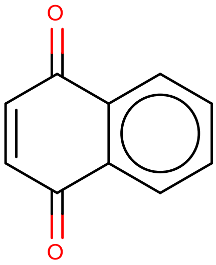	Naphthalene-1,4-dione	8/10	0.8
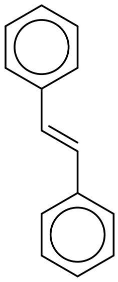	(E)-stilbene	28/34	0.82

Scaffold	Name	Positive Compounds /All Compounds	Ames Positive Rate
**Non-mutagenic Scaffolds**			
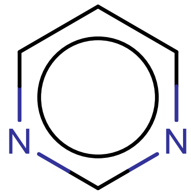	Pyrimidine	4/15	0.27
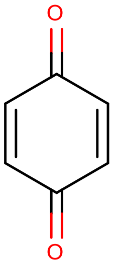	p-Benzoquinone	3/14	0.21
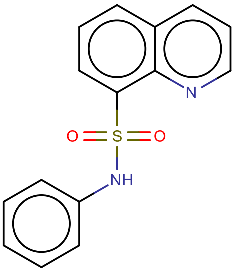	**(39)** N-phenyl-quinoline-8-sulfonamide	0/11	0
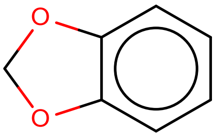	1,3-benzodioxole	2/14	0.14
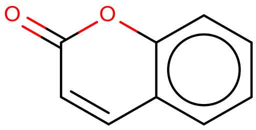	Coumarin	1/10	0.1

Scaffold	Name	Positive Compounds /All Compounds	Ames Positive Rate
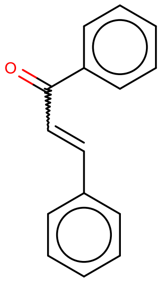	Chalcone	4/17	0.24
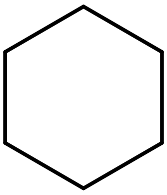	Cyclohexane	3/26	0.12
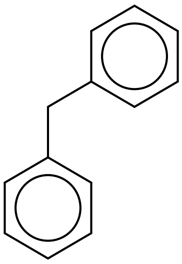	Diphenylmethane	16/49	0.33
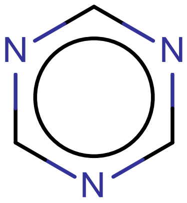	**(40)**<br>1,3,5-triazine	0/11	0
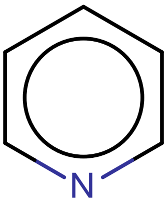	Pyridine	14/50	0.28

**Scaffold**	**Name**	**Positive Compounds /All Compounds**	**Ames Positive Rate**
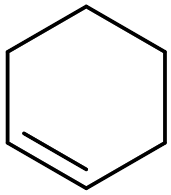	Cyclohexene	2/16	0.13
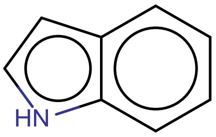	1H-indole	8/26	0.31

Figure A. The example of non-mutagenic compounds having the structural alert of "Polycyclic Aromatic Hydrocarbons".

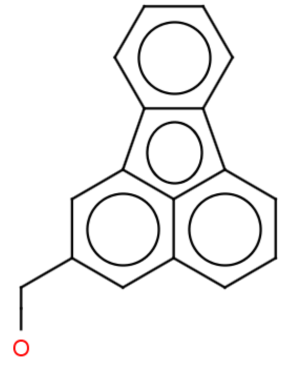

(34) fluoranthen-2-ylmethanol

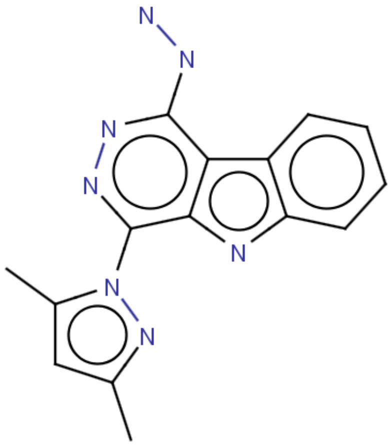

(35)
1-{1-hydrazinyl-3H-pyridazino[4,5-b]indol-4-yl}-3,5-dimethyl-1H-pyrazole

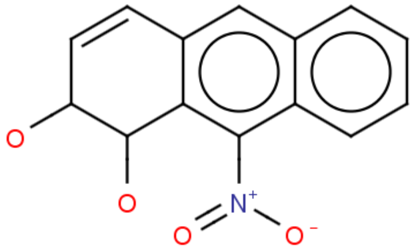

(36) 9-nitro-1,2-dihydroanthracene-1,2-diol

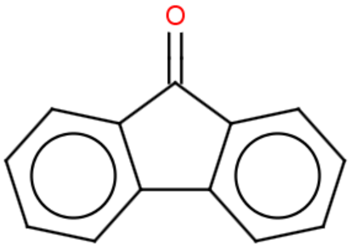

(37) 9H-fluoren-9-one

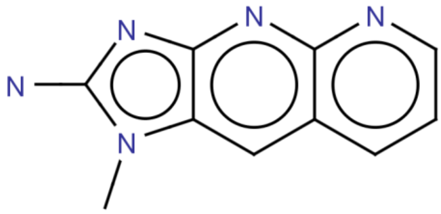

(38)
1-methyl-1H-imidazo[4,5-b]1,8-naphthyridin-2-amine

Figure B. The non-mutagenic compounds containing the non-mutagenic scaffolds of N-phenyl-quinoline-8-sulfonamide **(39)**, and 1,3,5-triazine **(40).**

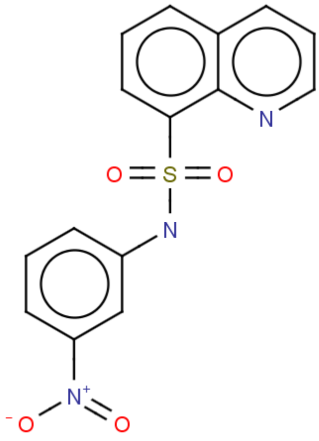

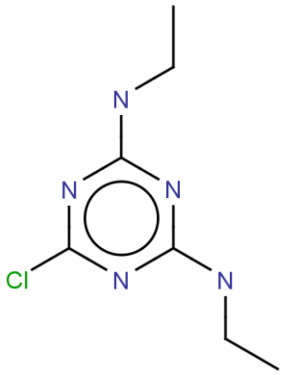

(41)

N-(3-nitrophenyl)quinoline-8-sulfonamide

(42)

6-chloro-2-N,4-N-diethyl-1,3,5-triazine-2,4-diamine

Figure C. The major scaffolds contributing to Ames mutagenicity.

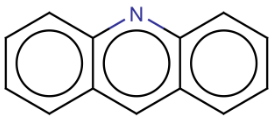

acridine

(0.94/53)

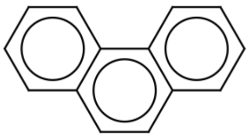

phenanthrene

(0.93/40)

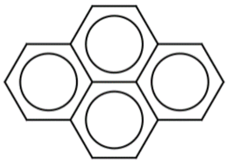

pyrene

(1/39)

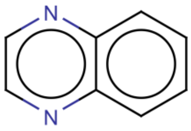

quinoxaline

(0.78/18)

Figure D. Scaffolds having mutagenic tendencies.

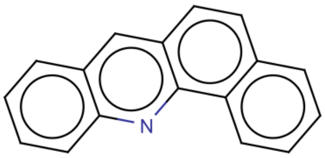

benzo[c]acridine

(0.86/21)

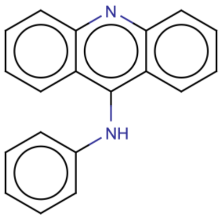

N-phenylacridin-9-amine

(0.94/18)

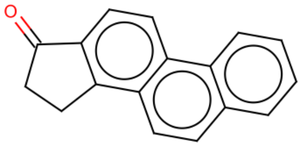

15,16-dihydrocyclopenta[a]phenanth ren-17-one

(0.77/13)

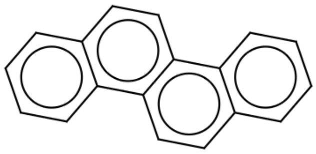

chrysene

(0.96/23)

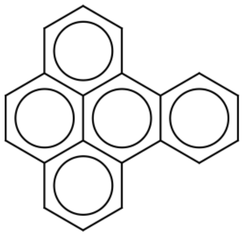

benzo[e]pyrene

(0.9/10)

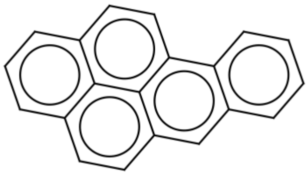

benzo[a]pyrene

(0.84/50)

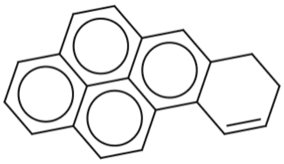

9,10-dihydro-benzo[a]pyrene

(0.9/10)

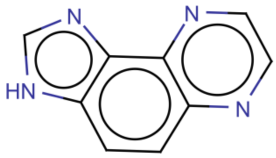

1H-imidazo[4,5-g] quinoxaline

(0.86/22)

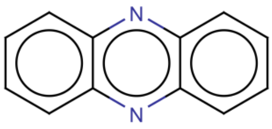

phenazine

(0.92/25)
